# Cashew nuts (*Anacardium occidentale* L.) decrease visceral fat, yet augment glucose in dyslipidemic rats

**DOI:** 10.1371/journal.pone.0225736

**Published:** 2019-12-12

**Authors:** Celina C. Q. Dias, Marta S. Madruga, Maria Manuela E. Pintado, Gabriel Henrique Oliveira Almeida, Ana Paula Vilar Alves, Francileide Amaro Dantas, Jéssyka Kallyne Galvão Bezerra, Marília Ferreira Frazão Tavares de Melo, Vanessa Bordin Viera, Juliana Késsia B. Soares

**Affiliations:** 1 DEA—Department of Food Engineering, Technology Centre, Federal University of Paraiba, João Pessoa, Paraiba, Brazil; 2 Universidade Católica Portuguesa, CBQF—Centro de Biotecnologia e Química Fina–Laboratório Associado, Escola Superior de Biotecnologia, Porto, Portugal; 3 Animal Science Departament, Federal University of Paraiba, Areia, Paraiba, Brazil; 4 Department of Nutrition, Center of Education and Health, Federal University of Campina Grande, Cuité, Paraíba, Brazil; University of Illinois, UNITED STATES

## Abstract

The objective of this study was to evaluate the biological effects of roasted Cashew nuts consumption on biochemical and murinometric parameters in dyslipidemic rats receiving lipid supplementation. Young male rats were randomly assigned to three experimental groups (n = 10). The Control group (CONT) was treated with water, the Dyslipidemic group (DL) received a high fat content emulsion throughout the experiment, and the Dyslipidemic Cashew Nuts group (DLCN) received the same high fat content emulsion throughout the experiment, yet was treated with Cashew nuts. Body parameters, biochemical, hepatic and fecal fatty acid profiles were all evaluated. The levels of total cholesterol and triglycerides were higher in the DL and DLCN groups as compared to the control group. DLCN and CONT presented no difference in HDL levels. DLCN presented higher glycemia levels than the other groups. There was reduction of body fat in DLCN as compared to other groups, but with higher accumulations of liver fat. DLCN presented a reduction in saturated hepatic fatty acids of 20.8%, and an increase of 177% in relation to CONT; there was also a 21% in increase DL for ω9 fatty acids in comparison to CONT. As for fecal fatty acids, there was a lower concentration of polysaturates in DLCN as compared to the other groups. The data showed that the consumption of Cashew nuts by the dyslipidemic animals treated with a hyperlipidic diet induced greater accumulations of liver fat and worsened glycemic levels, despite having reduced visceral fats and increased fecal fat excretion.

## Introduction

Oleaginous consumption in the population has increased considerably in recent years with worldwide growth estimated at 59% for the consumption of seed oils during the last decade. The most popular oleaginous nuts are Almonds (*Prunus amigdalis*), Hazelnuts (*Corylus avellana*), Pecans (*Juglans regia*), Brazil nuts (*Bertholletia excelsa*), Cashews (*Anacardium occidentale*), Pistachios (*Pistachia vera*), Pine nuts (*Pinus pinea*) and Macadamia nuts (*Macadam integrifolia*) [1].

Studies have evidenced the benefit of seed oils for human health [1], in particular the cholesterol-lowering effect [2], as well as cardioprotective effects of almonds [3], and reduction of inflammatory markers promoted by consumption of nuts in general [4]. Maternal consumption of Cashew nuts in rats has been investigated for causing alterations in offspring development [5], from acceleration of nervous system maturation, to prevention memory deficits. However, there are still few studies investigating the biological effects of Cashew nut seed oil in non-healthy populations.

Cashews are the fruit of the cashew tree (*Anacardium occidentale*), which when dried or roasted, originate Cashew nuts (also known as Cashews). Cashews are a tropical fruit native to northeastern Brazil, produced in large scale in India and Vietnam [6, 7] with global production from 2015 to 2016 reaching 738,861 tons [8]. Statistical data show a 70% increase in Cashew nut exports originating in these countries. The USA is the principal importer, but the population with the highest per capita consumption is Cambodia [8].

Cashew nuts are consumed in their natural or roasted form, or converted into food by-products [7]. Having a soft and slightly sweet flavor, they stand out for high lipid content (47.8 g/100g) as source of unsaturated fatty acids (UFAs)—oleic (ω-9) and linoleic (ω-6) acid [9, 5]. Other functional properties of the seed oil are due to its phenolic contents (flavonoids, anthocyanins and tannins), and fiber [10]. The most valuable micronutrients found in cashews are folate and tocopherols [11], which delay metabolic disorders, protecting against atherosclerosis and other chronic non-communicable diseases (CNCD) [12].

Cardiovascular disease is the most prevalent of CNCDs and responsible for almost 1/3 of deaths worldwide. The main risk factors for CNCDs are obesity, smoking, hypertension and dyslipidemias [13]. Adequate food habits are essential to control dyslipidemia; both fiber and unsaturated fatty acids help control dyslipidemia. Cashew nuts is a source of fiber and UFAs could be a food that helps control dyslipidemia [14]. We hypothesize that consumption of cashew nuts improves dyslipidemia in rats with a hyperlipidic diet. The objective of this research was to evaluate biological effects on biochemical and murinometric parameters of consuming cashew nuts in dyslipidemic rats who did not modify the hyperlipidic diet to normolipidic diet.

## Methods and materials

**Fig 1:**
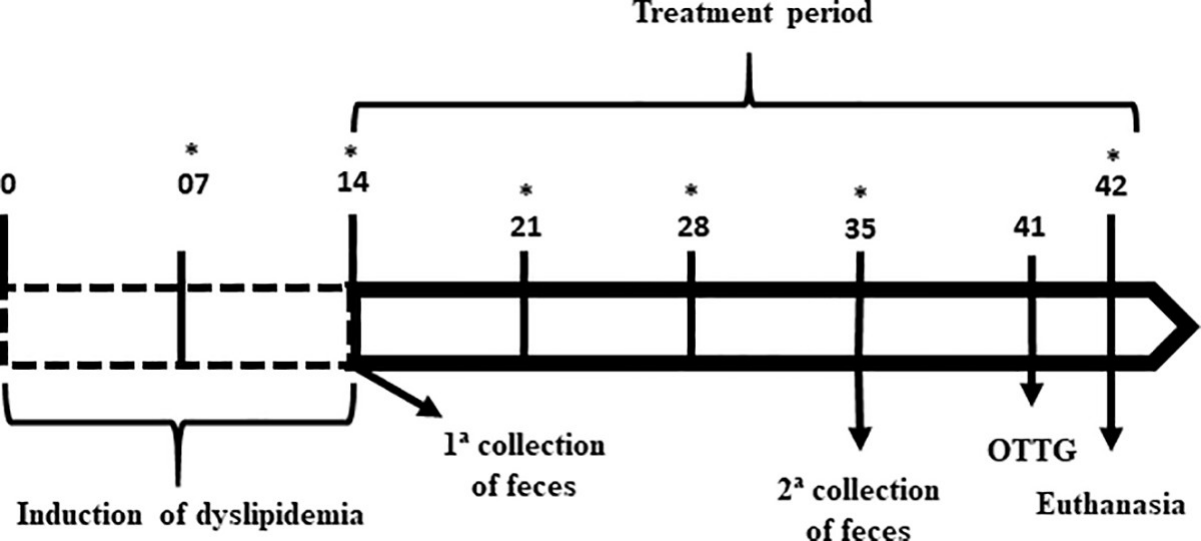
Experimental protocol. Sequence of time in days specifying procedures that were performed with dyslipidemic rats fed with cashew nuts, dyslipidemic control group and normal control group. (*) Days of monitoring of body weight and feed intake. Physical parameters, blood collection, adipose tissue and liver were performed on the same day of euthanasia.

This study was approved by the UFCG Ethics Committee for Animal Use (Protocol No. 94–2017). The experimental protocol followed the ethical recommendations of the National Institute of Health Bethesda (Bethesda, USA) regarding animal care (Fig 1). The research was duly registered in the National System of Management of Genetic Heritage and Traditional Knowledge (SISGEN), under Code A1BE84C.

### Plant material (cashew nuts)

Roasted cashew nuts were acquired in a local store in the city of Cuité, located in the Curimataú region of Paraíba. For better administration of the Cashew nuts to the animals, the Cashew nuts were crushed to flour. For this, the Cashew nuts were chilled to (± 4°C) to avoid temperature increases during processing; and loss of nutritional properties. A household blender was used to obtain the homogeneous flour which was then stored under refrigeration in a hermetically sealed container and unexposed to luminosity throughout the experiment.

### Nutritional characterization of the cachew nuts

Cashew nut flour was subjected to analysis to characterize its centesimal composition, fiber quantification, phenolic contents and total flavonoids (Table 1), as also analysis of phenolic compounds by High-performance liquid chromatography (HPLC) (Table 2). For centesimal composition; moisture, ash, lipids and proteins [15] were analyzed. Calculation of the total carbohydrate content was estimated by difference: using the AOAC formula [15]: 100-[Weight in grams (protein + lipids + ashes + water) in 100 g of food]. To quantify dietary fiber, the methodology described by Prosky et al. [16]. All analyses were performed in three replications and the results presented as mean and standard deviation.

**Table 1:** Centesimal composition from the diet and cashew nut.

Nutrient (g/Kg)	Diet (commercial chow)	Cashew nut
Carbohydrates	630	349.2 ± 0.0
Fat	40	372.3 ± 23.4
Protein	230	210.2 ± 7.3
Moisture	40	41.9 ± 0.7
Dry matter	60	26.4 ± 0.2
Total fibers	-	36.531 ± 0.0
Soluble fibers	-	3.311 ± 0.0
Insoluble fibers	-	33.220 ± 0.0
Total phenolics (mg GAE/100g)	-	80.20 ± 0.001
Total flavonoids (mg CE/100g)	-	6.90 ± 0.73

**Table 2:** Profile of phenolic components by HPLC present in cashew nuts.

Name	Amount (mg/100 g of cashew nut degreased)
***Phenolic acids***	
3,4-dihydroxy benzoic	200.78 ± 2.68
P-coumaric	10.71 ± 1.78
Syringic	20.52 ±6.25
Trans-Cinnamic	2.68 ± 0.89
Vanillic	29.45 ± 4.46
Ferulic	12.49 ± 0.00
Ellagic	59.79 ± 8.03
Caffeic	41.05 ± 3.57
Gallic	11.60 ± 0.89
***Flavonoids***	
Rutin	49.97 ± 7.14
Miricetin	37.48 ± 1.78
Quercetin	6.25 ± 0.89
Catechin	1650.82 ± 203.45
**Total**	2133,57

Values are expressed as mean ± SEM.

For evaluation of total phenolic and total flavonoids, the Cashew nut constituents were extracted with an 80% methanol solution (v/v). One gram of Cashew nut was measured into a test tube and 10 mL of solvent was added. The test tube was left at room temperature for 24 hours and after filtration the volume was completed to 10 mL with an extraction solvent and stored in a freezer (-18°C) until analysis. Total phenolic compounds were quantified according to the methodology described by Liu et al. [17], with modifications: 250 μL of extract was mixed with 1250 μL of a 1:10 diluted Folin–Ciocalteau reagent. The solutions were mixed thoroughly and incubated at room temperature (27°C) for 6 min. After incubation, 1000 μL of 7.5% sodium carbonate (Na_2_CO_3_) solution was added and again incubated in a water bath at 50°C for 5 min. The reaction mixtures’ absorbances were measured at 765 nm using a spectrophotometer (BEL Photonics, Piracicaba, São Paulo, Brazil). The absorbance of the extract was compared with a gallic acid standard curve for estimating concentration of total phenolic compounds in the sample. The results were expressed in mg of gallic acid equivalents (GAE) per hundred grams of Cashew nut on the basis of dry weight.

The total flavonoid content was measured using the colorimetric assay developed by Zhishen et al. [18]. A known volume (0.5 mL) of the extract was added to a test tube and at the same time 150 μL of 5% NaNO_2_ was added. After 5 min, 150 μL of 10% AlCl_3_ was added, and, at 6 min, 1 mL of 1 M NaOH was added, followed by 1.2 mL of distilled water. Sample absorbance was read at 510 nm using a spectrophotometer (BEL Photonics, Piracicaba, São Paulo, Brazil), and for estimating the concentration of flavonoid contents in the sample, it was compared with a catechin standard curve. The flavonoids content was expressed as mg of catechin equivalents (QE) per hundred grams of Cashew nut on the basis of dry weight.

The extraction, identification and quantification of phenolic acids from the cashew nuts were performed according to Meireles (19). For this, the milled sample was degreased with chloroform and methanol, ethanol 70% was added in proportion sample:solvent 1:10, stirred for 4h at 200 rpm under temperature control (26° C), centrifuged for 15 minutes at 5000 rpm. Then it was vacuum filtered with Buchner funnel. The extract was dried in a circulating air oven and eluted in water at a concentration of 5mg/mL and then injected into HPLC following the methodology mentioned [19].

### Animals and diets

Seven-week-old Wistar rats were randomly separated into three experimental groups (n = 10). Throughout the experiment all the animals were offered access (*ad libitum*) to water and commercial chow (Presence Purina ®), being housed individually in metabolic cages, in rooms with a 12 hours light/dark cycle, light phase starting at 6:00 a.m., with environmental temperature from 22 to 25°C, and relative humidity of ± 65%. The experimental groups formed were: the control group (CONT), receiving water by gavage; the Dyslipidemic Group (DL), receiving emulsion with high lipid content by gavage; and the Dyslipidemic Cashew Nut Group (DLCN), receiving an emulsion with high lipid content and Cashew nut flour by gavage.

The animals of the DL and DLCN groups underwent induction of dyslipidemia through administration of a lipid emulsion during the initial 14 days of the experiment, in the amount of 1 mL/100 g of the animal's weight, according to the methodology described by Xu et al. [20]. The emulsion contained pork lard, cholesterol, glycerol, propylthioracyl, and distilled water. After the initial 14 days, the formulation of the lipid emulsion was altered by removing the propylthioracyl and reducing the total quantity by half to 0.5 mL/100 g. Administrations continued to the DL and DLCN groups until the end of the experiment. Together with the lipid emulsion, the DLCN group animals received 1 g (4 g/kg of animal weight) of Cashew nut flour for 28 days.

### Physical parameters

Weight and feed intake verifications were performed weekly. The calorie intake was calculated from feed, emulsion with high lipid content intake and cachew nuts intake. At the end of the experiment, with the animal anesthetized, the nasal-anal length, and the abdominal and thoracic circumferences were measured by measuring tape. The body mass index (BMI) of the animals was calculated from the body weight (p) and nasal-anal length (c) data using the formula: BMI = p/c^2^, being weight (p) in grams, and length (c) in centimeters [21].

### Biochemical parameters

At the end of the experiment, the blood samples were obtained after cardiac puncture with the animals anesthetized using ketamine hydrochloride + xilasine hydrochloride (1 mL/kg of weight). Plasma was collected by centrifugation of the blood at 3,500 rpm for 15 min. The plasma was used to quantify total cholesterol (TC), HDL, triglycerides (TG) and blood glucose using the enzyme kit (LABTEST), with later spectrophotometer reading (Spectrophotometer SP 1102).

### Oral glucose tolerance test

The oral glucose tolerance test (OGTT) was performed at the end of the experiment on the 41^st^ experimental day. The animals at 6 hours of fasting received a 10% sucrose solution at 2 mL/100 g of weight. Blood was collected through the caudal vein. Glycemia was verified using an AccuCheck Active glucometer (Roche Diagnostics GmbH, Germany) at 0, 15, 30 and 45 minutes after administration of the solution.

The glucose area under the curve (AUC) were calculated as follows:

$$AUC(mg\;h/dL)=\frac{PG\;(0)+PG\;(15)\;x\;2+PG\;(30)\;x\;3+PG\;(45)\;x\;2}{4}$$

Where, PG is plasma glucose.

### Visceral, retroperitoneal and hepatic fats

Shortly after euthanasia, the visceral (mesenteric and epididymal) and retroperitoneal fats were removed and weighed [22]. The liver was removed, weighed and subjected to fat quantification by means of the methodology described by Folch et al. [23], and beginning from a 2 g lipid extraction of the sample using 40 mL of a chloroform: methanol (2:1) solution.

### Fecal fat

The feces of the animals were collected in two periods of the study. The first collection took place at the end of dyslipidemia induction on the 14^th^ experimental day, and the second was performed at the end of the third week of treatment—on the 35^th^ experimental day. The Folch et al. [23] methodology was used for fecal fat quantification.

### Hepatic and fecal fatty acids

Parts of the liver and feces samples from the animals were used for fatty acid quantification. Methylation of the fatty acids present in the lipid extract was carried out for both, following the methodology described by Hartman and Lago [24]. An aliquot of the lipid extract was taken, calculated for each sample according to the fat content found in the lipid measurement; and quantification was performed according to the method of Folch et al. [23]. Adding 1 mL of internal standard (C19:0) and a saponification (KOH) solution, the solution was subsequently heated under reflux for 4 min. An esterification solution was then added immediately afterwards, returning the solution to heating under reflux for 3 extra minutes. The sample was then allowed to cool before subsequent washings with ether, hexane, and distilled water; finally obtaining an extract (with the methyl esters and solvents), which was conditioned into a properly identified amber glass until complete drying of the solvents. After drying, a suspension was made (in 1 mL of hexane) and packaged into a vial for further chromatographic analysis. The aliquots of the saponification and esterification solutions were determined according to the methodology described by Hartman and Lago [24].

A gas chromatograph (VARIAN 430-GC, California, USA), coupled to a fused silica capillary column (CP WAX 52 CB, VARIAN, California, USA) with dimensions of 60 m x 0.25 mm, and a 0.25 mm film thickness was used with helium as the carrier gas (1 mL/min flow rate). The initial temperature was 100°C programmed to reach 240°C, increasing 2.5°C per minute for 30 min, totaling 86 minutes. The injector temperature was maintained at 250°C and the detector at 260°C. 1.0 μL aliquots of esterified extract were injected in a Split/Splitless injector. The chromatograms were recorded using *Galaxie Chromatography Data System software*. Fatty acids were identified by comparing the retention times for the methyl esters of the samples against Supelco Mix C4-24/C19 standards. The fatty acid results were quantified by normalizing the areas of the methyl esters, and are expressed as percentage of area.

### Statistical analysis

The Cashew nuts flour composition result was described as mean ± S.D. All other results were expressed as mean ± S.E.M. The statistical analysis of the data was based on one-way ANOVA followed by a Tukey’s test. Differences were considered significant when p < 0.05. The statistical analyses were performed using the GraphPad Prism 7 statistical software.

## Results

### Phenolic compounds

The identification and quantification by HPLC of cashew nut phenolic components is shown in Table 2. Thirteen phenolic compounds were identified among phenolic acids and flavonoids. The highest concentrations were found in 3,4-dihydroxy benzoic acids (200.78 ± 2.68), Ellagic acid (59.79 ± 8.03) and catechin (1650.82 ± 203.45).

### Body weight

**Fig 2:**
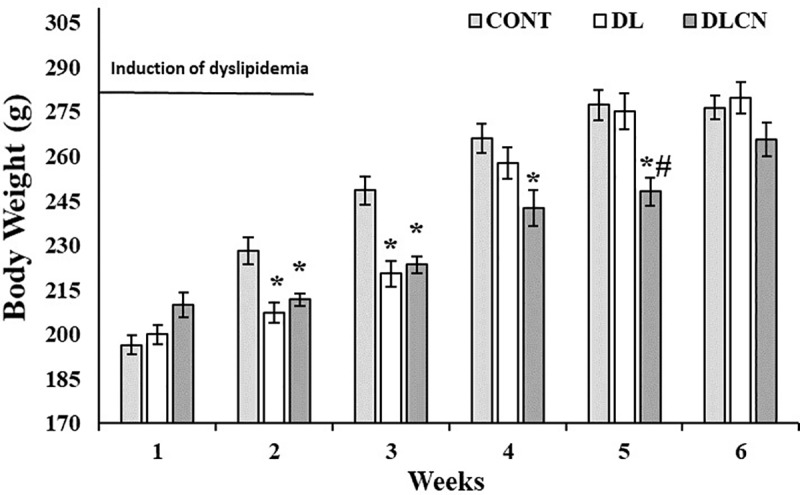
Weekly body weight of rats with a hyperlipidaemic diet treated with cashew nut. Values are the mean ± SEM. Control group (CONT), Dyslipidemic group (DL), Dyslipidemic Cashew Nut group (DLCN). * *versus* CONT. **#** *versus* DL. p < 0.05, as determined by One-Way ANOVA followed by the Tukey test.

As for the weekly weight of the animals in the respective second and third weeks of the experiment, it was found that the groups undergoing the dyslipidemic induction and treatment transitions (DL 207.5 ± 3.44 g; 220.5 ± 4.31 g) and (DLCN 211.75 g ± 2.02; 223.6 ± 2.87 g) presented lower weights in relation to the control group (CONT 228.2 ± 4.61 g; 248.6 ± 4.73 g) (P < 0.05). In the fourth week, the reduction in weight continued only for the DLCN (242.6 ± 5.89 g) in relation to the CONT (266.0 ± 4.88 g), and in the fifth week, the DLCN (248.2 ± 4.75 g) also presented lower weights as compared to the other two experimental groups, CONT (277.3 ± 5.12 g), and DL (275.14 ± 6.05 g) (P < 0.05) (Fig 2).

### Food and calories intake

**Fig 3:**
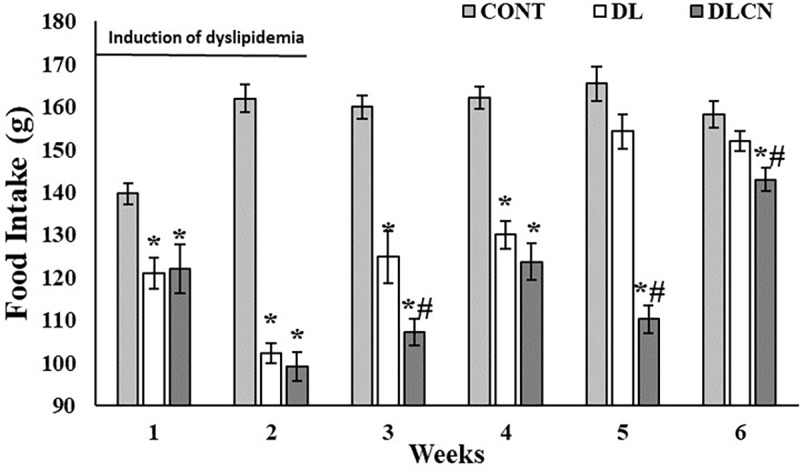
Weekly feed intake of dyslipidemic rats treated with cashew nut. Values are the mean ± SEM for the following groups: Control group (CONT), Dyslipidemic group (DL), Dyslipidemic Cashew Nut group (DLCN). * *versus* CONT. **#** *versus* DL. p < 0.05, as determined by One-Way ANOVA followed by the Tukey test.

During dyslipidemia induction, the DL (121.0 ± 3.65 g; 102.3 ± 2.35 g) and DLCN (122.0 ± 5.67 g; 99.12 ± 3.44 g) groups consumed less ration as compared to CONT (139.7 ± 2.45 g; 162.0 ± 3.20 g) (P < 0.05). In the third and fourth week the DL group (124.8 ± 6.06 G; 130.0 ± 3.18 g) consumed even less ration as compared to CONT (160.0 ± 2.73 G; 162.2 ± 2.65 g). In the fourth week of the experiment the DLCN (110.2 ± 4.26 g) also presented lower feed intake in relation to CONT (162.2 ± 2.65 g), and in the other weeks consumed less ration than the other groups as well (P < 0.05) (Fig 3).

The caloric intake presented in Table 3 shows that CONT consumed more calories from the high feed intake compared to the other groups in the first experimental weeks. In the last two experimental weeks, DLCN consumed fewer calories compared to the other groups.

**Table 3:** Calorie intake (Kcal) of dyslipidemic rats treated with cashew nuts.

Week	CONT	DL	DLCN
1	530.86 ± 9.30^a^	471.19 ± 13.99^b^	483.02 ± 21.63^ab^
2	615.60 ± 12.15^a^	399.01 ± 8.93^b^	382.73 ± 15.43^b^
3	608.00 ± 10.36^a^	485.45 ± 23.28^b^	423.98 ± 12.36^b^
4	618.86 ± 10.07^a^	506.87 ± 14.48^b^	488.05 ± 16.42^b^
5	628.90 ± 15.18^a^	593.39 ± 15.79^a^	436.72 ± 12.36^b^
6	601.35 ± 11.88^a^	591.34 ± 9.24^a^	547.38 ± 4.83^b^

Values are expressed as mean ± SEM. Different letters in the same row differ significantly (p < 0.05) between the samples.

### BMI, abdominal and thoracic circumferences

In Table 4, the BMI, abdominal and thoracic circumference values of the experimental groups are described. No significant differences were observed between the groups (P > 0.05).

**Table 4:** Physical parameters of dyslipidemic rats treated with cashew nuts.

Parameters	CONT	DL	DLCN	P value
Body mass index (g/cm^2^)	0.527 ± 0.02	0.575 ± 0.01	0.568 ± 0.02	0.154
Abdominal circumference (cm)	17.49 ± 0.25	17.2 ± 0.39	16.45 ± 0.28	0.060
Thoracic circumference (cm)	15.72 ± 0.29	15.05 ± 0.47	14.72 ± 0.27	0.150

Values are expressed as mean ± SEM.

### Biochemical analysis

The biochemical analyses proved the efficacy of the dyslipidemia inductions since both the DL (69.59 ± 4.39 mg/dl) and DLCN (122.52 ± 12.95 mg/dl) groups had high levels of total cholesterol in relation to the CONT (43.72 ± 2.47 mg/dl) (P < 0.05). DLCN also presented higher values than DL (P < 0.05). The DL group presented higher serum triglyceride values (127.4 ± 12.56 mg/dl) as compared to CONT, while DLCN (81.56 ± 5.26 mg/dl) presented elevated triglycerides in relation to CONT (62.76 ± 6.24 mg/dl) and reduced triglycerides as compared to DL (P < 0.05).

HDL levels were similar for the group treated with Cashew nuts and the control group, but were higher in the DLCN (48.17 ± 3.53 mg/dl) than in the DL (29.55 ± 2.89 mg/dl) (P < 0.05). In the DL group, the HDL content was lower than in the CONT group (45.28 ± 5.46 mg/dl) (P < 0.05).

**Fig 4:**
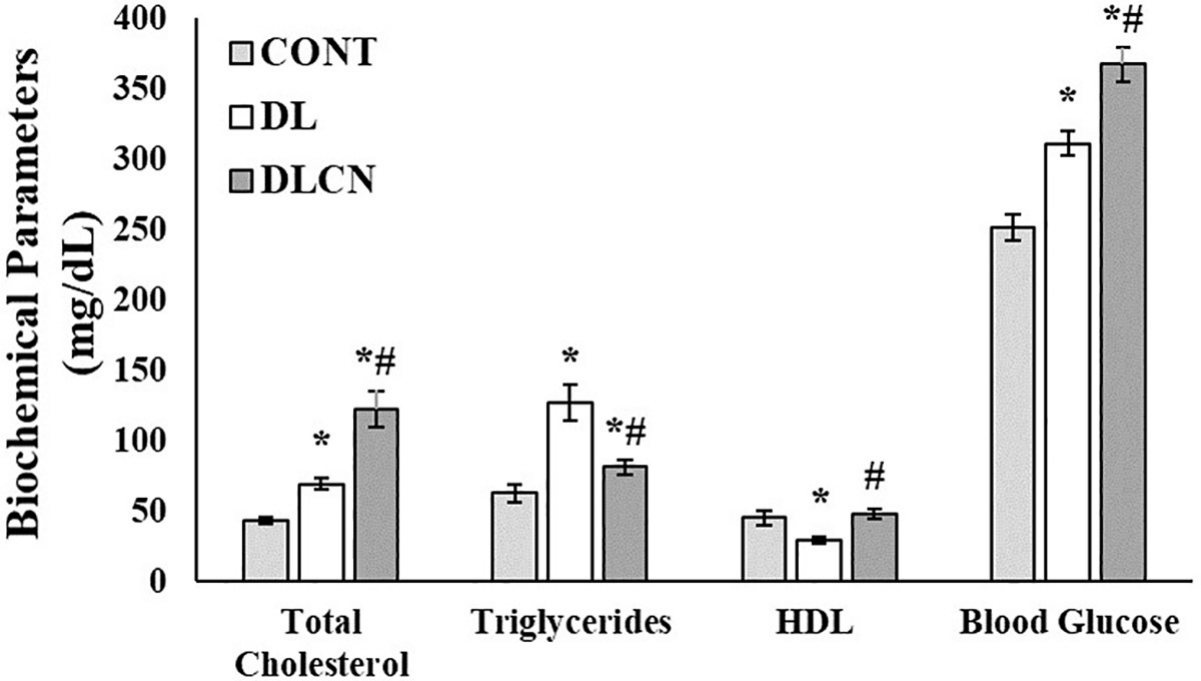
Biochemical values (mean ± SEM) of dyslipidemic rats fed cashew nut. CONT = Control group, DL = Dyslipidemic group, DLCN = Dyslipidemic Cashew Nut group. * *versus* CONT, **#** *versus* DL; For all groups, p < 0.05 was considered a significant difference as determined by One-Way ANOVA followed by the Tukey test.

Regarding glycemic levels, DLCN (367.65 ± 12.48 mg/dl) presented higher values than the other groups (DL 311.39 ± 8.59 mg/dl, and CONT 251.54 ± 9.56 mg/dl). However, the DL group also presented elevated glycemia as compared to CONT (P < 0.05) (Fig 4).

### Oral tolerance test for glucose (OTTG)

**Fig 5:**
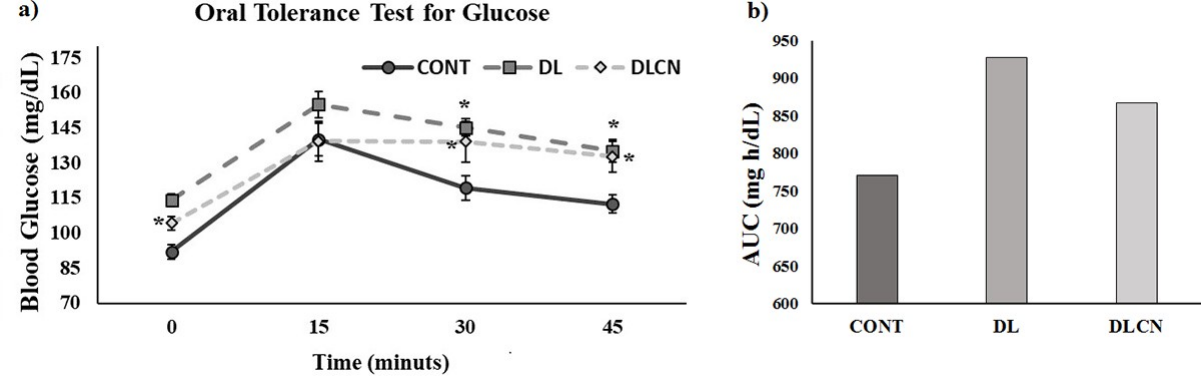
Glycaemic curve of oral tolerance test for glucose for dyslipidaemic rats treated with cashew nut and glucose area under the curve. a) Glycaemic curve of oral tolerance test for glucose; b) glucose area under the curve (AUC). Values are the mean ± SEM. Groups: CONT = Control group; DL = Dyslipidemic group; DLCN = Dyslipidemic Cashew Nut group. * *versus* CONT. p < 0.05, as determined by One-Way ANOVA followed by the Tukey test.

During OTTG, the animals of the DLCN (104.20 ± 2.97 mg/dl; 132.78 ± 6.05 mg/dl) and DL (114.11 ± 2.83 mg/dl; 135.1 ± 4.93 mg/dl) groups had higher glycemic peaks in relation to CONT (92.0 ± 3.18 mg/dl; 112.5 ± 3.76 mg/dl), respectively at times 0 (zero) and 45 min. At 30 min, only DL (145.11 ± 3.68 mg/dl) presented an increase as compared to CONT (119.33 ± 5.25 mg/dl) (P < 0.05) (Fig 5A).

Still analyzing the Fig 5, we can follow the OTTG results through the glucose area over the curve (Fig 5B), showing higher glucose levels for the DL group (926.99 mg h/dL), followed by the DLCN (866.91 mg h/dL) compared to CONT (771.04 mg h/dL).

### Visceral, retroperitoneal and hepatic fats

**Fig 6:**
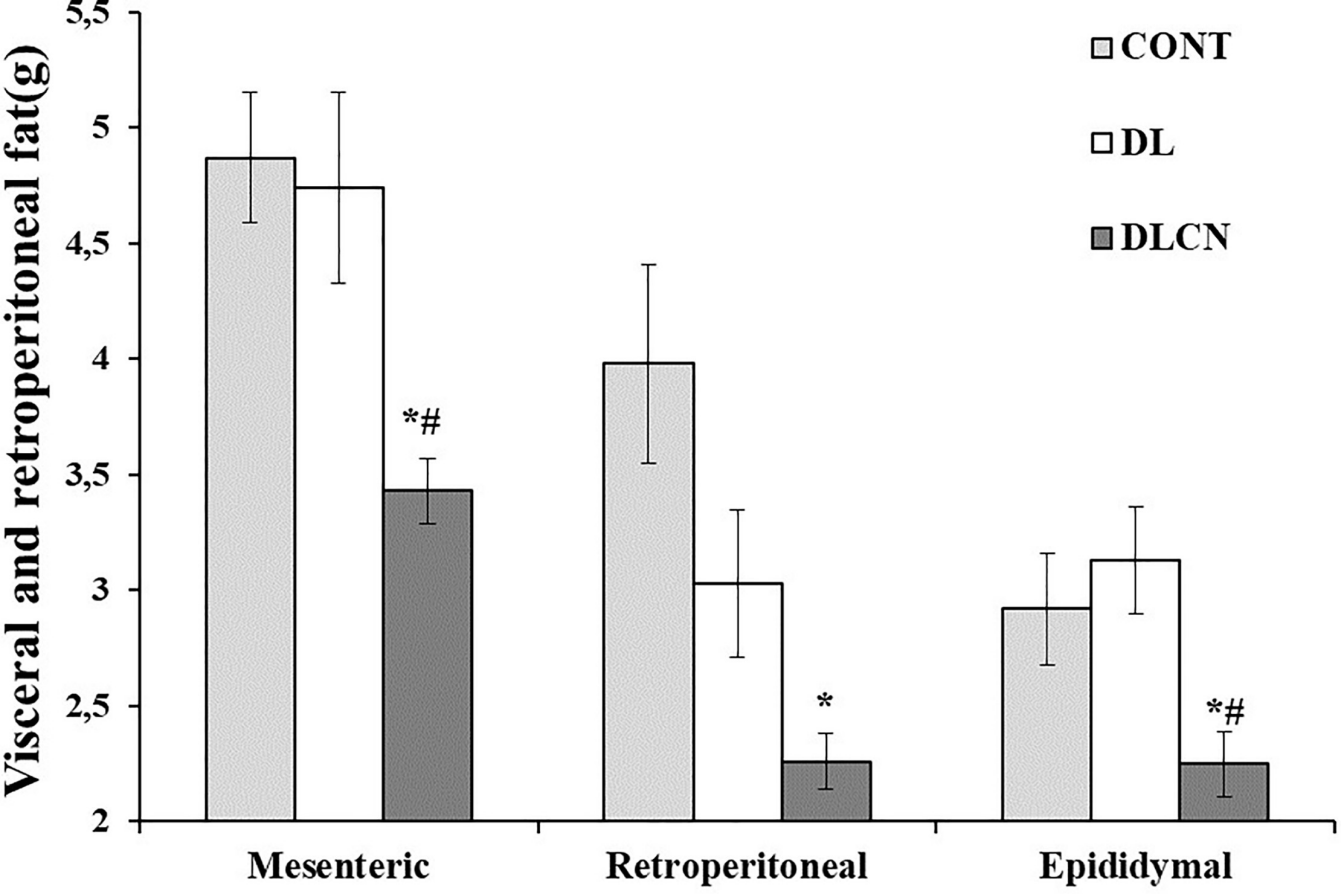
Weight of visceral fat (mesenteric and epididymal) and retroperitoneal of dyslipidaemic rats fed cashew nut. Values are the mean ± SEM. Control group (CONT), Dyslipidemic group (DL), Dyslipidemic Cashew Nut group (DLCN). * *versus* CONT. # *versus* DL. p < 0.05, as determined by One-Way ANOVA followed by the Tukey test.

**Fig 7:**
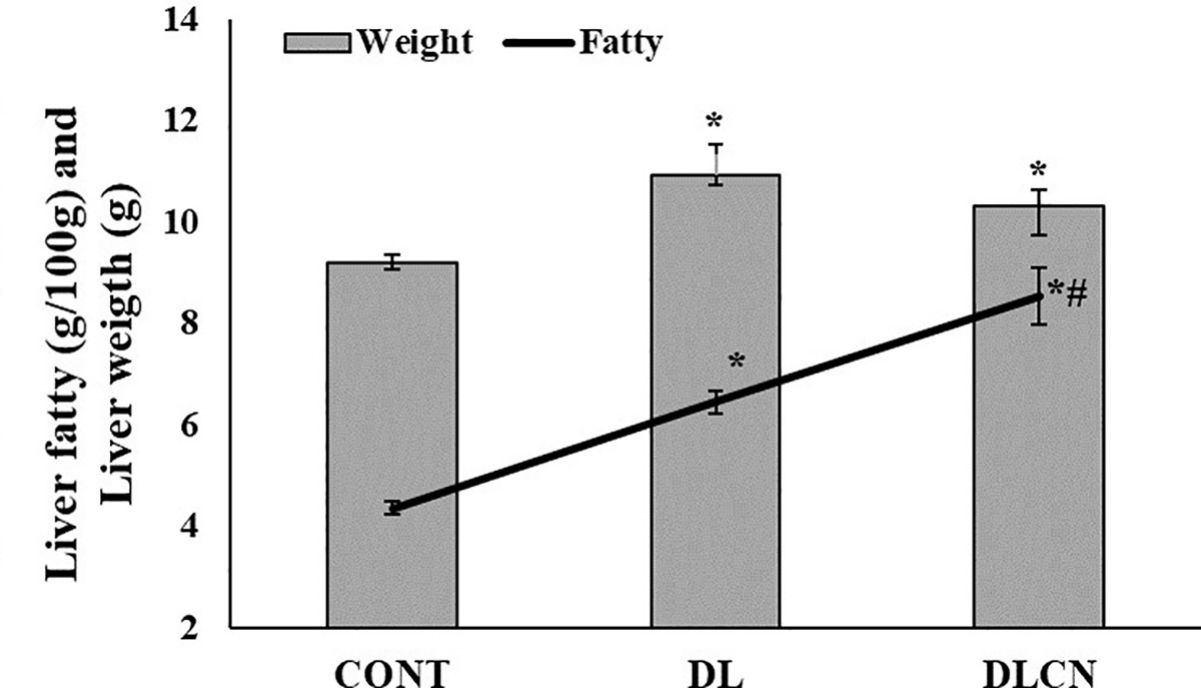
Liver fatty and liver weight of dyslipidaemic animals fed cashew nut for four weeks. Values expressed as the mean ± SEM. The groups were Control group (CONT), Dyslipidemic group (DL), Dyslipidemic Cashew Nut group (DLCN). * *versus* CONT. **#** *versus* DL. p < 0.05, as determined by One-Way ANOVA followed by the Tukey test.

The measurements of visceral fats showed that DLCN accumulated less mesenteric and epididymal fat respectively (3.43 ± 0.14 g; 2.25 ± 0.14 g) in relation to the other groups (CONT 4.87 ± 0.28 g; 2.92 ± 0.24 g and DL 4.74 ± 0.41 g; 3.13 ± 0.23 g), and in the retroperitoneal space (2.26 ± 0.12 g) when compared to CONT (3.98 ± 0.43 g) (P < 0.05) (Fig 6). The liver weight differed between the DLCN (10.33 ± 0.32 g) and DL (10.95 ± 0.58 g) groups as compared to the CONT (9.2 ± 0.17 g) (P < 0.05) (Fig 7).

The quantification of liver fat showed that the DLCN (8.55 ± 0.57 g/100g) accumulated more fat than the CONT (4.36 ± 0.12 g/100g) and DL (6.45 ± 0.22 g/100g) groups (P < 0.05). However, the DL group accumulated a higher hepatic fat content than the CONT group (Fig 7).

### Fecal fat

**Fig 8:**
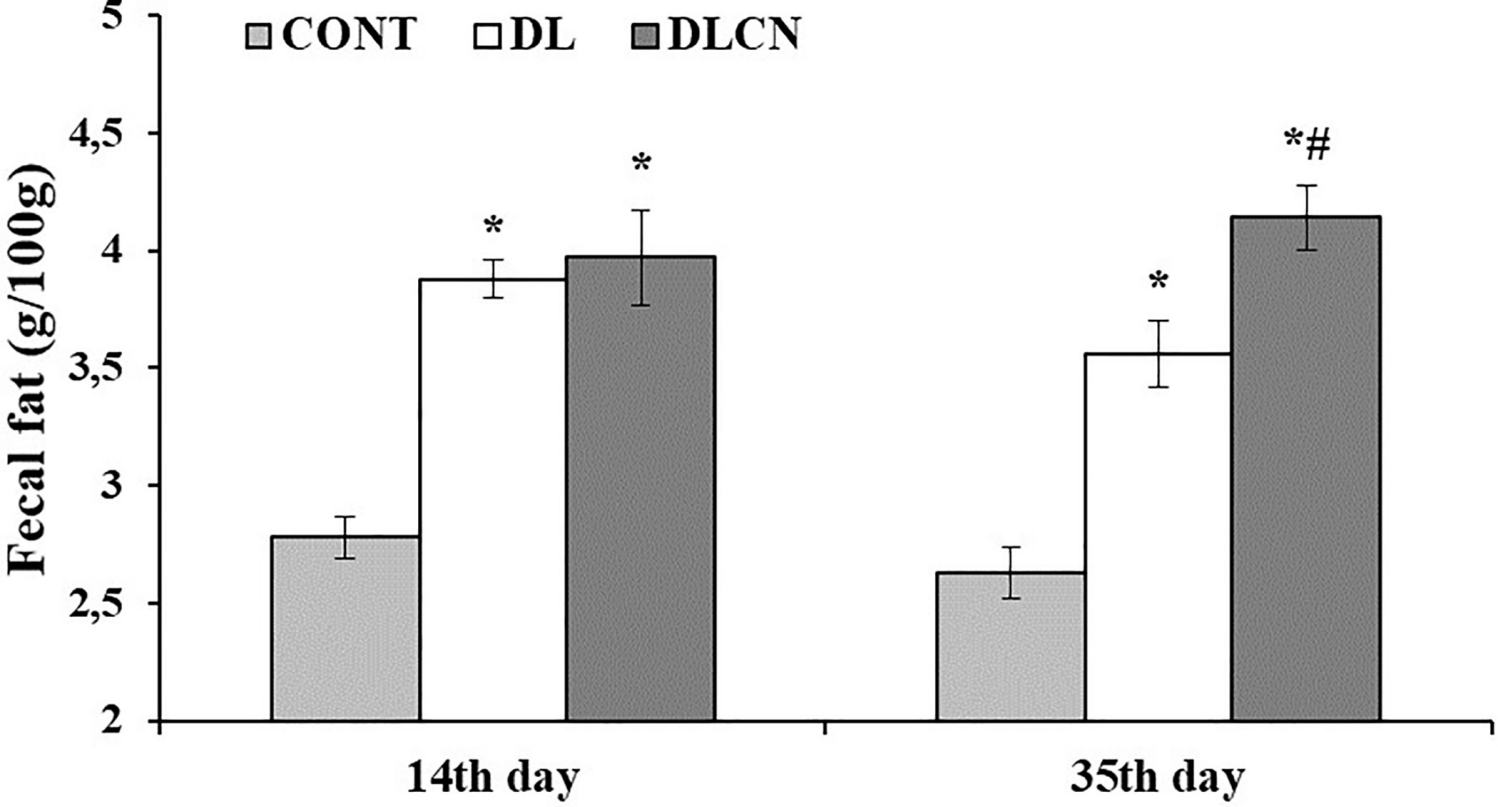
Fecal fat of rats treated with a high-fat diet with cashew nut. Rats were divided into the following groups: Control group (CONT), Dyslipidaemic group (DL) and Dyslipidemic Cashew Nut group (DLCN). The first collection occurred at 14^th^ day of the experiment and the second collection at 35^th^ day of experiment. * *versus* CONT. # *versus* DL, with p < 0.05, determined by One-Way ANOVA followed by the Tukey test, where values are expressed as the mean ± SEM.

The fat content in the feces was higher in the DL (3.88 ± 0.08 g/100g; 3.56 ± 0.14 g/100g) and DLCN (3.97 ± 0.20 g/100g; 4.14 ± 0.14 g/100g) groups in relation to CONT (2.78 ± 0.09 g/100g; 2.63 ± 0.11 g/100g) for all collections. However, in the second collection, the DLCN group (4.14 ± 0.14 g/100g) excreted more fat compared to the other groups (DL 3.56 ± 0.14 g/100g) and CONT (2.63 ± 0.11 g/100g), as illustrated in Fig 8.

### Hepatic and fecal fatty acids

Regarding saturated fatty acids (SFA) present in the liver (Table 5), the DLCN group presented an increase in the content of Myristic acid (14:0) as compared to DL and a reduction as compared to CONT. There was a reduction of Palmitic acid (16:0) in the DLCN group compared to the other groups; the highest value was in the DL group. Stearic acid (18:0) resulted in similar values for DLCN and DL, with lower values than CONT. The DLCN group presented higher values of Lignoceric acid (24:0) compared to DL, but with no difference from CONT. In total values of accumulated SFA in the hepatic tissue, the DLCN group presented a reduction of 20.8% as compared to the CONT group, and of 1.5% as compared to the DL group.

**Table 5:** Composition of fatty acids (%) present in the liver of rats fed with cashew nuts.

FATTY ACIDS	GROUPS		
	CONT	DL	DLCN
**SATURATED**			
Myristic acid **14:0**	5.02 ± 0.25	2.62 ± 0.26*	4.50 ± 0.30*^#^
Pentadecanoic acid **15:0**	0.11 ± 0.01	0.05 ± 0.01*	0.05 ± 0.01*
Palmitic acid **16:0**	15.65 ± 0.20	16.35 ± 0.28*	13.70 ± 0.00*^#^
Margaric acid **17:0**	0.41 ± 0.03	0.29 ± 0.00*	0.31 ± 0.01*
Stearic acid **18:0**	19.05 ± 1.05	13.05 ± 0.14*	13.14 ± 0.02*
Arachidic acid **20:0**	0.03 ± 0.00	0.04 ± 0.00	0.07 ± 0.00*^#^
Behenic acid **22:0**	0.05 ± 0.01	0.07 ± 0.02	0.12 ± 0.02*^#^
Lignoceric acid **24:0**	0.23 ± 0.01	0.13 ± 0.03*	0.22 ± 0.02^#^
**TOTAL**	**40.55**	**32.60**	**32.11**
**MONOUNSATURATED**			
Myristoleic acid **14:1ω5**	0.15 ± 0.01	0.12 ± 0.02	0.16 ± 0.01^#^
Palmitoleic acid **16:1ω7c**	0.46 ± 0.01	0.96 ± 0.10*	1.00 ± 0.02*
Heptadenoic acid **17:1ω7c**	0.18 ± 0.02	0.13 ± 0.06	0.16 ± 0.03
Trans-Oleic acid **18:1ω9t**	0.14 ± 0.00	0.22 ± 0.02*	0.43 ± 0.03*^#^
Cis-Oleic acid **18:1ω9c**	8.53 ± 0.04	19.85 ± 0.14*	24.23 ± 0.06*^#^
Gondoic acid **20:1ω9**	0.05 ± 0.01	0.27 ± 0.07*	0.23 ± 0.04*
Erucid acid **22:1ω9**	0.71 ± 0.01	1.33 ± 0.03*	1.41 ± 0.00*^#^
Nervonic acid **24:1ω9**	0.12 ± 0.01	0.12 ± 0.02	0.16 ± 0.02*^#^
**TOTAL**	**10.34**	**23.00**	**27.78**
**POLYUNSATURATED**			
Linoleic acid **18:2ω6c**	14.91 ± 0.24	20.62 ± 0.23*	18.10 ± 0.02*^#^
γ-linolenic acid **18:3ω6**	0.15 ± 0.04	0.15 ± 0.04	0.13 ± 0.05
α-linolenic acid **18:3ω3**	0.09 ± 0.01	0.23 ± 0.07*	0.13 ± 0.06^#^
Eicosadienoic acid **20:2ω6**	0.26 ± 0.03	0.52 ± 0.01*	0.42 ± 0.01*^#^
Eicosatrienoic acid **20:3ω6c**	0.11 ± 0.00	0.06 ± 0.01*	0.05 ± 0.02*
Eicosatrienoic acid **20:3ω3c**	0.05 ± 0.03	0.06 ± 0.00	0.06 ± 0.01
Arachidonic acid **20:4ω6c**	22.53 ± 0.60	14.56 ± 0.09*	14.33 ± 0.05*
Docosadienoic acid **22:2ω6**	0.35 ± 0.19	0.34 ± 0.08	0.29 ± 0.13
Eicosapentaenoic acid **20:5ω3**	0.40 ± 0.08	0.56 ± 0.03*	0.21 ± 0.05*
Docosahexaenoic acid **22:6ω3**	3.89 ± 0.17	3.53 ± 0.05*	2.81 ± 0.05*^#^
**TOTAL**	**42.74**	**40.63**	**36.53**
**PUFA/SFA**	**1.05**	**1.25**	**1.14**
**Σ ω3**	**4.43**	**4.38**	**3.21**
**Σ ω5**	**0.15**	**0.12**	**0.16**
**Σ ω6**	**38.31**	**36.25**	**33.32**
**Σ ω7**	**0.64**	**1.09**	**1.16**
**Σ ω9**	**9.55**	**21.79**	**26.46**
**ω6/ω3**	**8.65**	**8.28**	**10.38**

Values are expressed as mean ± SEM. CONT = Control Group; DL = Dyslipidemic Group; DLCN = Dyslipidemic Cashew Nut Group; Σ = sum; PUFA = Polyunsaturated Fatty Acids; SFA = Saturated Fatty Acids.

* *vs* CONT.

^#^ *vs* DL. Statistical test used was One-Way ANOVA followed by Tukey with level of significance (p <0.05).

As for the amount of monounsaturated fatty acids (MUFA) present in the liver, DLCN presented a higher value of Myristoleic acid (14:1ω5) as compared to DL. DLCN also presented higher values of Trans-oleic acid, Cis-oleic acid, Erucid and Nervonic acids (18:1ω9t, 18:1ω9c, 22:1ω9 and 24:1ω9) in relation to the other groups. The sum of the MUFA averages showed an increase of 168% for the DLCN group in relation to the CONT group and 21% in relation to the DL group. The total content of ω-9 fatty acids was higher in the DLCN group as compared to the other groups, with an elevation of 177% in relation to CONT, and 21% in relation to LD.

The polyunsaturated fatty acid Linoleic acid (18:2ω6c) presented elevated values (by about 17% for DLCN) when compared to CONT, and a higher quantification in the DL group as compared to the other groups. Arachidonic acid (20:4ω6c) presented similar values for the DLCN and DL groups, with a reduction in the CONT group. The fatty acid Eicosapentaenoic acid (20:5ω3) presented increasing values for DLCN, CONT and DL in order. The Docosahexaenoic acid (22:6ω3) values were lower in the DLCN group and higher in the CONT group. The total polyunsaturated fatty acids (PUFA) in the liver presented a slight percentage difference between the groups, with DLCN presenting a 14.5% reduction as compared to CONT, DL presenting a 4.9% reduction as compared to CONT, and DLCN presenting a 10% reduction as compared to DL.

Our data show in Table 6 that fecal excretion of fatty acids varied between groups in both feces collections. The data from the first collection, performed on the last day of dyslipidemia induction, show an increase in saturated fats for the groups that consumed the emulsion with high fat content. However, in the second collection there was a marked decrease in saturated fat excretion by the DLCN group as compared to the other groups, and a difference of 10.6% was observed in relation to the DL group. MUFA quantification presented similar levels between the two collections and between the groups. Regarding PUFA, there was a reduction in the second collection as compared to the first for the CONT and DL groups, but the DLCN presented lower levels of polyunsaturated fats in the feces for both the first and second collections in relation to the other groups.

**Table 6:** Composition of fatty acids (%) present in the feces of dyslidemics rats fed with cashew nuts.

FATTY ACIDS	GROUPS					
	CONT		DL		DLCN	
	14^th^ day	35^th^ day	14^th^ day	35^th^ day	14^th^ day	35^th^ day
**SATURATED**						
Myristic acid **14:0**	8.47 ± 0.02	9.19 ± 0.28	10.80 ± 0.33*	9.39 ± 0.60	9.99 ± 0.56*^#^	10.85 ± 0.84*^#^
Pentadecanoic acid **15:0**	1.70 ± 0.14	3.11 ± 0.24	1.26 ± 0.25*	2.16 ± 1.13*	1.95 ± 0.18^#^	2.61 ± 0.25*^#^
Palmitic acid **16:0**	16.02 ± 0.13	16.97 ± 0.24	14.97 ± 0.06*	15.78 ± 0.36*	16.42 ± 0.18*^#^	14.63 ± 0.19*^#^
Margaric acid **17:0**	0.10 ± 0.01	0.24 ± 0.02	0.05 ± 0.03*	0.16 ± 0.06	0.19 ± 0.02*^#^	0.35 ± 0.12^#^
Stearic acid **18:0**	15.24 ± 0.34	9.95 ± 0.20	15.25 ± 0.13	16.78 ± 0.01*	16.04 ± 0.15*^#^	11.35 ± 0.32*^#^
Arachidic acid **20:0**	0.73 ± 0.03	1.05 ± 0.04	0.52 ± 0.04*	0.97 ± 0.16	0.49 ± 0.08*	0.63 ± 0.14*^#^
Behenic acid **22:0**	0.65 ± 0.01	1.01 ± 0.04	0.40 ± 0.04*	0.65 ± 0.00*	0.52 ± 0.00*^#^	0.43 ± 0.00*^#^
Lignoceric acid **24:0**	0.99 ± 0.03	1.91 ± 0.02	0.79 ± 0.05*	1.23 ± 0.07*	0.74 ± 0.01*	1.27 ± 0.16*
**TOTAL**	**43.9**	**43.43**	**44.04**	**47.12**	**46.34**	**42.12**
**MONOUNSATURATED**						
Myristoleic acid **14:1ω5c**	2.41 ± 0.01	2.35 ± 0.15	1.91 ± 0.23*	1.88 ± 0.17*	2.22 ± 0.20^#^	2.61 ± 0.04*^#^
Pentadecenoic acid **15:1ω5c**	0.33 ± 0.03	0.49 ± 0.00	0.19 ± 0.05*	0.33 ± 0.03*	0.20 ± 0.03*	0.43 ± 0.02*^#^
Palmitoleic acid **16:1ω7c**	0.50 ± 0.06	0.58 ± 0.00	0.42 ± 0.11	0.62 ± 0.09	0.61 ± 0.09^#^	0.66 ± 0.17
Oleic acid **18:1ω9t**	2.39 ± 0.05	3.64 ± 0.11	1.82 ± 0.01*	2.12 ± 0.14*	2.06 ± 0.01*^#^	0.97 ± 0.18*^#^
Oleic acid **18:1ω9c**	16.36 ± 0.25	15.13 ± 1.02	16.04 ± 1.41	14.59 ± 0.97	15.92 ± 0.28	16.50 ± 0.05*^#^
Erucid acid **22:1ω9**	0.18 ± 0.05	0.28 ± 0.06	0.18 ± 0.02	0.20 ± 0.00*	0.15 ± 0.07	-
**TOTAL**	**22.17**	**22.47**	**20.56**	**19.74**	**21.16**	**21.17**
**POLYUNSATURATED**						
Linoleic acid **18:2ω6c**	20.69 ± 0.05	19.34 ± 0.32	18.92 ± 0.28*	17.84 ± 0.79*	18.17 ± 0.27*^#^	17.48 ± 0.18*^#^
γ-linolenic acid **18:3ω6**	0.33 ± 0.08	0.16 ± 0.05	0.23 ± 0.02*	0.18 ± 0.08	0.26 ± 0.02*	0.16 ± 0.03
α-linolenic acid **18:3ω3**	0.82 ± 0.00	0.73 ± 0.00	1.04 ± 0.00*	0.84 ± 0.00*	0.57 ± 0.00*^#^	0.39 ± 0.00*^#^
Eicosadienoic acid **20:2ω6**	0.17 ± 0.01	0.15 ± 0.05	0.19 ± 0.02	0.29 ± 0.04*	-	-
Arachidonic acid **20:4ω6c**	0.26 ± 0.00	0.26 ± 0.01	0.48 ± 0.07*	0.41 ± 0.02*	0.43 ± 0.08*	0.35 ± 0.09*
Eicosapentaenoic acid **20:5ω3**	0.46 ± 0.05	0.41 ± 0.03	0.76 ± 0.07*	0,57 ± 0.01*	0.50 ± 0.03^#^	0.76 ± 0.00*^#^
Docosahexaenoic acid **22:6ω3**	0.36 ± 0.14	0.62 ± 0.05	0.77 ± 0.07*	0.48 ± 0.06*	0.64 ± 0.03*	1.08 ± 0.14*^#^
**TOTAL**	**23.09**	**21.67**	**22.39**	**20.61**	**20.57**	**20.22**
**PUFA/SFA**	**0.52**	**0.50**	**0.51**	**0.44**	**0.44**	**0.47**
**Σ ω3**	**1.64**	**1.76**	**2.57**	**1.89**	**1.71**	**2.23**
**Σ ω5**	**2.74**	**2.84**	**2.10**	**2.21**	**2.42**	**3.04**
**Σ ω6**	**21.45**	**19.91**	**19.82**	**18.72**	**18.86**	**17.99**
**Σ ω7**	**0.50**	**0.58**	**0.42**	**0.62**	**0.61**	**0.66**
**Σ ω9**	**18.93**	**19.05**	**18.04**	**16.91**	**18.13**	**17.47**
**ω6/ω3**	**13.08**	**11.31**	**7.71**	**9.90**	**11.03**	**8.07**

Values are expressed as mean ± SEM. The first collection occurred at 14^th^ day of the experiment and the second collection at 35^th^ day of experiment. CONT = Control group; DL = Dyslipidemic Group; DLCN = Dyslipidemic Cashew Nut Group; Σ = sum; PUFA = Polyunsaturated Fatty Acids; SFA = Saturated Fatty Acids.

* *vs* CONT.

^#^ *vs* DL. Statistical test used was One-Way ANOVA followed by Tukey with level of significance (p <0.05).

## Discussion

Consumption of seed oils (pleasant flavors and high caloric values) is related to body weight gain [25]; but the fiber content of this food also acts on satiety, causing hypophagy, and consequent reduction of ingested energy [26]. Increased energy density in a diet with high lipid value, such as Cashew nut consumption with an emulsion rich in SFA and cholesterol as used in the present study also causes increased satiety [27], with consequent reductions in the appetites of the rats [28]. These findings justify the results of the present study in relation to the delay in weight gain and lower calories and feed intake by the groups that underwent induction of dyslipidemia (Table 3, Figs 2 and 3). However, in the DLCN animals, the result was even more evident, since the consumption of Cashew nuts caused lower calories and feed intake as compared to the other experimental groups, most evident in the last experimental weeks (Table 3 and Fig 3).

When assessing appetite sensations in humans ingesting almonds and walnuts, levels of hunger were found to be suppressed and satiety increased [29, 30]. Like almonds and other nuts, Cashew nuts present high lipid and protein contents, as well as fibers which are associated with reduced appetite [29].

The supplementation of fibers in rats fed a diet rich in fats has also caused a decrease in animals’ weights, but with increased consumption [31, 28]. Another factor associated with alteration of satiety is the profile and amount of fatty acids in the lipid source consumed; these eventually flow through the bloodstream and affect satiety in the brain. After triacylglycerol hydrolysis of foods, fatty acids are transported to epithelial cells in the form of micelles to be absorbed. Depending on the degree of fat insaturation, micelle formation becomes faster; UFAs are more readily available for absorption, causing release of hormones and increased satiety [32].

Kozimor et al. [33], evaluated satiety in women receiving a diet rich in SFA, MUFA, and PUFA and it was observed that PUFAs promoted greater satiety in comparison to SFAs. In the study, MUFAs presented a lower satiety response as compared to the other two types of fat. Poudyal and collaborators [34] concluded that supplementation with differing oils such as: “macadamia oil, safflower oil, and linseed oil, being respectively rich in oleic, linoleic, and α-linolenic acids, reduced feed intakes as compared to groups fed a normal diet. A study comparing a diets rich in saturated and unsaturated fats, found higher weights in rats fed a diet rich in saturated fats, even with the same calorie intake for both groups [35].

The Cashew nuts used in this research presented excellent total phenolic content and total flavonoids; as well as a high catechin content, seen in Tables 1 and 2, giving it a high nutritional quality [36]. According to Trox et al. [37], Cashew nuts present high antioxidant potential because they are a source of phenolic compounds which possess biological and medicinal properties. Like other oleaginous foods, the total phenolic content and total flavonoids found in Cashew nuts are highlighted for their functional properties [38, 39], being responsible for inhibiting or reducing oxidation, and resulting in UFA protection [40]. However, Cashew nuts’ antioxidant content can be diminished through heat treatment, percentage of shell present, storage, and irradiation processes [40]. Studies show the potential value of *Anacardium occidentale* as a source of antioxidants not only in the fruit, but also in its leaves and pseudofruits [41, 42].

The induction of dyslipidemia as performed in the present study proved to be effective, since the treatment induced an increase in TC in both treated groups (DL and DLCN) (Fig 4). However, the consumption of Cashew nuts did not reverse the effects caused by the administration of a high fat content emulsion. The Cashew nuts used in the present study presented a total dietary fiber content of 3.65 g per 100 g of product, (with 0.33 g of soluble and 3.32 g of insoluble fibers). Soluble fibers are widely used in the treatment of dyslipidemias [43, 44]. In contrast, insoluble fiber consumption does not present positive effects for cholesterol reduction or cardiovascular risk [14]; as verified in the present study, because there were no observed reductions in TC or TG.

The hypocholesterolemic action caused by consumption of seed oils still diverges in many studies. Lovejoy et al. [45], evaluated non-diabetic adult males and females who consumed 100 g of almonds/day for 4 weeks. A reduction in TC by 21% was observed. Similarly, Lee et al. [46] reported improvement of TC levels in women with metabolic syndrome who consumed a nut mixture for 6 weeks. However, Casas-Agustench et al. [26], when assessing the effect of a seed oil mixture (walnuts, almonds and hazelnuts), in conjunction with a standard diet in adult men and women with metabolic syndrome observed (after 12 weeks) no alterations in TC, LDL, HDL and TG. When evaluating the administration of Cashew leaf, stem and nut extract, Jaiswal et al. [47], reported no significant differences in TC and HDL levels in diabetic rats.

In the present study, the HDL levels in the dyslipidemic group decreased in comparison to the control group. However, in the animals treated with Cashew nuts, which also suffered prior induction of dyslipidemia, the damage was reversed. The DLCN presented values similar to the control group (which did not suffer from induction of dyslipidemia) (Fig 4), thus highlighting the beneficial effects of Cashew nuts on HDL levels.

Evaluating the glycemic metabolism of the animals in the present study through fasting glycemia, the group treated with Cashew nuts presented higher values as compared to the other groups (Fig 4). In the oral glucose tolerance test, the groups DL and DLCN presented elevated levels of serum glucose at the beginning and end of the test (time 0, 30 and 45 minutes) (Fig 5A), this can be confirmed in Fig 5B with the glucose area over the OTTG curve. However, the curve shows that DL had higher serum glucose levels. Studies evaluating seed oil consumption in humans have observed a reduction in fasting glycemia in diabetic individuals [48, 49, 45]. However, Ma et al. [50], when treating diabetic men and women with nuts, verified an increase in fasting glycemia.

Research also performed with humans presents results similar to the present study when performing the OGTT, confirming an increase in serum glycemia in men and women diagnosed with metabolic syndrome and treated with Cashew nuts for 8 weeks [51]. When testing a diet with high fat content, Almeida-Suhett et al. [52], also found an increase in basal glycemia in rats, resulting in a glucose tolerance curves with greater areas in the animals fed the high fat content diets.

Regarding serum TG levels, DLCN presented a decrease of 36.3% in relation to the dyslipidemic group and a 23% elevation as compared to the control group. Because they are a source of UFA, Cashew nuts promote a fall in TG because they potentially reduce exposure of non-esterified fatty acids to the liver, preventing one of the main TG synthesis pathways [53]. However in this study, Cashew nuts were not able to completely ablate such alterations as compared to the control group.

Alterations in carbohydrate metabolism interfere in the lipid profile, because when glucose appears in excess, insulin converts it into fatty acids which are stored in the form of TG, (their concentration becomes elevated and HDLs are reduced). Elevation of TG levels implies pancreatic β cell apoptosis due to lipotoxicity, causing insulin resistance [54]. Thus, elevation of serum TG levels correlates with high fasting glucose levels and the OGTT results in this study. The induction of dyslipidemia and the consumption of Cashew nuts may have triggered increased glycemic levels which interfere with lipid metabolism.

A diet rich in lipids, such as the one used in the present study, causes the organism to accumulate excess fat, with adipocyte expansion, high blood concentrations of serum lipids and lipoproteins, and high lipid supplies to the liver. Lipid superaccumulation results in the ectopic deposition of lipids in non-adipose tissues, and increases hepatic gluconeogenesis; along with LDL and HDL reductions [55, 56].

In our study, the animals of the DL and DLCN groups were kept on a fat-rich diet, and the DLCN group received Cashew nuts (in addition) which also have high lipid content. The data revealed that supplementation with Cashew nuts induces a lower deposition of fatty acids in the adipose tissues, besides promoting a decrease in serum TG. The data correlate when considering that TG are stored in the adipose tissue and that deposition is directly related to its synthesis in the liver and its concentration in the bloodstream [57].

However, reduction in fat deposition was not observed in the liver, since there was a higher accumulation of fats in the DLCN than in the DL. Evaluating the fatty acids profile as accumulated in this tissue, a difference was observed; the group that received Cashew nuts presented more MUFA in relation to the DL group.

Considering that the DLCN group received fatty acids (originating in Cashew nuts) in addition to the dietary fatty acids which the DL group also received. Cashew nut supplementation inhibited fat accumulation in adipose tissue as compared to DL. However, when analyzing the glycemic curve we also found an increase in plasma glycemia. According to Kahn and Valdez [55], a fat-rich diet can generate free fatty acid deposition in the pancreas; reduce insulin secretion, inducing insulin resistance, or hyperglycemia. This potentially occurred in the present study, since there was an increase in the glycemic curve and in fasting hyperglycemia.

When the physical condition of the animals was evaluated, it was found that the Cashew nuts offered to the animals caused (besides reduction of body weight) a reduction of visceral (mesenteric and epididymal) and retroperitoneal fats (Fig 6). Vaidya et al. [58] evaluated the effect of omega 3 fatty acid supplementation in rats with a fat-rich diet, and observed reductions in body fat in animals treated for 8 weeks. The same research observed reduction of body weight, reduction of liver weight, reduction of TC, and of hepatic cholesterol. Bhaskaran et al. [35], when treating rats with saturated and unsaturated fats found a reduction in the size of the adipocytes of the group fed with a diet rich in unsaturated fat. These studies corroborate our findings, where Cashew nuts, a source of unsaturated fats, produced the same results.

Fat-rich diets also induce adipose tissue accumulation; which causes the increase of free plasmatic fatty acids [59]. The release of free fatty acids from visceral adipose tissues contributes to fat oxidation, and stimulates esterification of fatty acids into TGs in the liver. This leads to reductions in glucose and lipid metabolism in the peripheral tissues [60]. Our research presented reductions of visceral and retroperitoneal fats in the group treated with Cashew nuts. In contrast, there was an increase in serum TG (as compared to the control group) and higher liver fat accumulation, which might be explained by increased oxidation of fatty acids and reduction of lipogenesis, which uses peripheral tissue fats [35].

The hepatic fat content measured followed the organ weight; since as fat deposition increases, there is an increase in organ weight. Our findings evidenced greater deposition of hepatic fat in the group treated with Cashew nuts as compared to the other groups (CONT and DL), but the liver weight increased in both dyslipidemic groups (DLCN and DL) as compared to CONT (Fig 7). Cholesterol and TG synthesis is highest in the liver, and the main sites of fat deposition are in the viscera and under the skin.

In this study, the animals that consumed Cashew nuts presented lower abdominal fat deposition. Both dyslipidemic groups presented a higher percentage of liver fat, with the highest amount found in DLCN. The correlation of hepatic fat deposition with quantification of fatty acids revealed that when comparing the DLCN with the DL, the amounts of SFA were similar; with the MUFA contents being 21% higher in the DLCN. These results are possibly due to Cashew nuts being a source of oleic acid. The consumption of Cashew nuts induced greater hepatic deposition of MUFA. As in our research, Picklo et al. [61], also found an increase in oleic acid in the livers of rats with a high-fat oleic acid diet; as well as an elevation of liver fats and glycemia for these animals.

High depositions of inappropriate ectopic fat in the liver generate hepatic steatosis or non-alcoholic fatty liver disease (NAFLD) [57], a disease with negative impacts on the health of obese individuals, and usually accompanied by dyslipidemia and other complications [58, 62].

Lee Homma and Fujii [63] showed that the early phase of non-alcoholic fatty liver disease (NAFLD) presents protective functions against oxidative lesions caused by reactive oxygen species (ROS) and toxic agents in rats when fed a diet rich in unsaturated fat, because this temporary accumulation of liver fat is an adaptive response of hepatocytes under stress. Research comparing the accumulation of liver fats as related to the consumption of SFA, (with a palm oil based diet), and UFA, (with sunflower oil-based diet) in adult men and women concluded that a UFA rich diet prevents liver, visceral, and total fat deposition as compared to the SFA rich diet. This was not observed in the present study [53].

The Cashew nuts used in this research are a source of oleic acid and linoleic acid, however, since it was offered to previously dyslipidemic animals and this continued with consumption of a hyperlipid emulsion, the Cashew nut consumption was not able to reverse liver fat accumulation or to improve the fatty acid profile in the tissue, with the exception of the MUFA.

In the first collection, the amount of fat excreted in the feces was higher in the DL and DLCN groups than in the CONT group, due to induction of dyslipidemia through administration of a high fat content emulsion. However, in the second collection, (after initiation of treatment with Cashew nuts), there was a higher amount of fat excretion by DLCN as compared to CONT and DL (Fig 8). The use of fibers in the diet assists in the excretion of fat through the feces [64]. Cashew nuts, in addition to containing dietary fibers, are a food source of UFAs, especially oleic and linoleic acids. High fat intake, (as in the case of the animals in the DL group), induced higher fecal fat excretion, and the consumption of Cashew nuts (which is a source of fibers) also caused an increase in excretion. The soluble fibers form a chelate with the fat being excreted by the gallbladder or consumed in the diet [65].

Thus, in the present study, the data confirm that the fibers and lipid content of Cashew nuts were responsible for the increase in fecal fat excretion. When using seed oils (walnuts, almonds and hazelnuts) in research with adult individuals, Casas-Agustench et al. [26] found higher excretion of fecal fat when compared to the control group. Research evaluating dietary fiber contents, found that a diet supplemented with flaxseed increased fat excretion by up to 50% [64]. Even with increased fat excretion, DLCN showed high total cholesterol, but this group consumed largely unsaturated cashew lipids, which may be responsible for increasing the HDL content compared to DL.

When correlating the data of this study, we found that Cashew nuts in a dyslipidemic diet (DLCN), in relation to the group without Cashew nuts (DL), led to reversal of HDL serum levels, weight reduction, visceral and retroperitoneal fat deposition, higher liver fat deposition (of a better quality) and increased excretion of fecal fat. However, we also found increased serum levels of total cholesterol, together with glucose.

## Conclusion

Previously dyslipidemic animals, maintained on saturated fats associated with Cashew nuts, presented reductions in visceral and retroperitoneal fat deposition, and a reversal of diminished HDL levels usually found in dyslipidemias. However, treatment with Cashew nuts compromised glycemic metabolism, and augmented fat deposition in the hepatic tissue.

We conclude that consumption of Cashew nuts by dyslipidemic animals in an unbalanced diet presents improvements in dyslipidemia, yet also increases glycemic alterations, and raises risks of non-alcoholic fatty liver disease.

## Supporting information

S1 Fig Experimental protocol. (TIF)

S2 Fig Chromatogram for the analysis of cashew nut phenolic compounds. (TIF)

S1 Table Commercial chow composition. (DOCX)

## References

[1] Grosso G, Estruch R. Nut consumption and age-related disease. Maturitas. 2016;84:11–16. 10.1016/j.maturitas.2015.10.014 26586104

[2] Sabaté J, Haddad E, Tanzman JS, Jambazian P, Rajaram S. Serum lipid response to the graduated enrichment of a Step I diet with almonds: a randomized feeding trial. Am J Clin Nutr. 2003;77:1379–84. 10.1093/ajcn/77.6.1379 12791613

[3] Albert CM, Gaziano JM, Willett WC, Manson JE. Nut consumption and decreased risk of sudden cardiac death in the Physicians' Health Study. Arch Intern Med. 2002;162:1382–87. 10.1001/archinte.162.12.1382 12076237

[4] Jiang R, Jacobs DR Jr, Mayer-Davis E, Szklo M, Herrington D, Jenny NS, et al Nut and seed consumption and inflammatory markers in the multi-ethnic study of atherosclerosis. Am J Epidemiol. 2006;163:222–31. 10.1093/aje/kwj033 16357111

[5] Melo MFFT, Pereira DE, Sousa MM, Medeiros DMF, Lemos LTM, Madruga MS, et al Maternal intake of cashew nuts accelerates reflex maturation and facilitates memory in the offspring. International Journal of Developmental Neuroscience. 2017;61:58–67. 10.1016/j.ijdevneu.2017.06.006 28663041

[6] Andrade TJAS, Araújo BQ, Citó AMGL, Silva J, Saffi J, Richter MF, et al Antioxidant properties and chemical composition of technical Cashew Nut Shell Liquid (tCNSL). Food Chemistry. 2011;126:1044–48.

[7] Carvalho ALN, Annonia R, Silva PRPS, Borelli P, Fock RA, Trevisan MTS, et al Acute, subacute toxicity and mutagenic effects of anacardic acids from cashew (Anacardium occidentale Linn.) in mice. Journal of Ethnopharmacology. 2011;135:730–36. 10.1016/j.jep.2011.04.002 21511024

[8] NUTS & DRIED FRUITS STATISTICAL YEARBOOK 2016/2017. International Nut and Dried Fruit Council Foundation (INC). Available im: < http://www.nutfruit.org/wp-continguts/uploads/2017/06/Statistical-Yearbook-2016-2017.pdf> Access date: 01/07/2017.

[9] Agila A, Barringer SA. Volatile Profile of Cashews (*Anacardium occidentale L*.) from Different Geographical Origins during Roasting. Journal of Food Science. 2011;76(5):768–74. 10.1111/j.1750-3841.2011.02180.x 22417425

[10] Baptista A, Gonçalves RV, Bressan J, Peluzio MCG. Antioxidant and Antimicrobial Activities of Crude Extracts and Fractions of Cashew (Anacardium occidentale L.), Cajui (Anacardium microcarpum), and Pequi (Caryocar brasiliense C.): A Systematic Review. Oxidative Medicine and Cellular Longevity. 2018;2018:13p. 10.1155/2018/3753562 29849888 PMC5932493

[11] Alexiadou K, Katsilambros N. Nuts: Anti-atherogenic food? European Journal of Internal Medicine. 2011;22:141–46. 10.1016/j.ejim.2010.11.008 21402243

[12] Gómez-Caravaca AM, Verardo V, Caboni MF. Chromatographic techniques for the determination of alkyl-phenols, tocopherols and other minor polar compounds in raw and roasted cold pressed cashew nut oils. Journal of Chromatography A. 2010;1217:7411–17. 10.1016/j.chroma.2010.09.054 20961547

[13] Wong ND. Epidemiological studies of CHD and the evolution of preventive cardiology. Nat. Rev. Cardiol. 2014;11:276–89. 10.1038/nrcardio.2014.26 24663092

[14] Faludi AA, Izar MCO, Saraiva JFK, Chacra APM, Bianco HT, Afiune Neto A, et al Atualização da Diretriz brasileira de dislipidemias e prevenção da aterosclerose. Arquivos Brasileiro de Cardilogia. 2017;109(2). 10.5935/abc.20170121 28813069

[15] AOAC INTERNATIONAL. Official methods of analysis. 16^th^ ed. Gaitherburg: Published by AOAC International 1997;2:1–43.

[16] Prosky L, Asp NG, Sceweizer TF. Determination of insoluble, soluble and total fiber in food products: interlaboratory study. J Assoc Off Anal Chem. 1988;71(5):1017–23. 2853153

[17] Liu M, Li XQ, Weber C, Lee CY, Brown J, Liu RH. Antioxidant and antiproliferative activities of raspberries. Journal of Agricultural and Food Chemistry. 2002;50:2926–30. 10.1021/jf0111209 11982421

[18] Zhishen J, Mengchen T, Jianming W. The determination of flavonoid contents in mulberry and their scavenging effects on superoxide radicals. Food Chemistry. 1999;64:555–59.

[19] Meireles BRLA. Potencial nutricional e antioxidante do fruto do catolé (S*yagrus cearensis*). M.Sc. Thesis, Universidade Federal da Paraíba. 2017. Available from: https://repositorio.ufpb.br/jspui/handle/123456789/12729

[20] Xu D, Xu M, Lin L, Rao S, Wang J, Davey AK. The effect of isosteviol on hyperglycemia and dyslipidemia induced by lipotoxicity in rats fed with high-fat emulsion. Life Sciences. 2012;90:30–38. 10.1016/j.lfs.2011.10.010 22075495

[21] Novelli ELB, Diniz YS, Galhardi CM, Ebaid GMX, Rodrigues HG, Mani F, et al Anthropometrical parameters and markers of obesity in rats. Laboratory Animals. 2007;41:111–119. 10.1258/002367707779399518 17234057

[22] Cinti S. The Adipose Organ. Prostaglandins, Leukotrienes and Essential Fatty Acids. 2005;73:9–15. 10.1016/j.plefa.2005.04.010 15936182

[23] Folch J, Lees M, Sloane Stanley GH. A simple method for the isolation and purification of total lipids from animal tissues. Journal Biological Chemistry. 1957;226:497–509. 13428781

[24] Hartman L, Lago RCA. Rapid preparation of fatty acid methyl from lipids. Lab. Pract. 1973;22:475 4727126

[25] Vadivel V, Kunyanga CN, Biesalski HK. Health benefits of nut consumption with special reference to body weight control. Nutrition. 2012;28:1089–97. 10.1016/j.nut.2012.01.004 23044160

[26] Casas-Agustench P, López-Uriarte P, Bulló M, Ros E, Cabré-Vila JJ, Salas-Salvadó J. Effects of one serving of mixed nuts on serum lipids, insulin resistance and inflammatory markers in patients with the metabolic syndrome. Nutrition, Metabolism & Cardiovascular Diseases. 2011;21:126–135. 10.1016/j.numecd.2009.08.005 20031380

[27] Neyrinck AM, Bindels LB, De Backer F, Pachikian BD, Cani PD, Delzenne NM. Dietary supplementation with chitosan derived from mushrooms changes adipocytokine profile in diet-induced obese mice, a phenomenon linked to its lipid-lowering action. International Immunopharmacology. 2009;9:767–773. 10.1016/j.intimp.2009.02.015 19286482

[28] Chang S, Cui X, Guo M, Tian Y, Xu W, Huang K, et al Insoluble Dietary Fiber from Pear Pomace Can Prevent High-Fat Diet-Induced Obesity in Rats Mainly by Improving the Structure of the Gut Microbiota. J. Microbiol. Biotechnol. 2017;27(4):856–867 10.4014/jmb.1610.10058 28173692

[29] Tan SY, Mattes RD. Appetitive, dietary and health effects of almonds consumed with meals or as snacks: a randomized, controlled trial. European Journal of Clinical Nutrition. 2013;67:1205–1214. 10.1038/ejcn.2013.184 24084509 PMC3898316

[30] Brennan AM, Sweeney LL, Liu X, Mantzoros CS. Walnut consumption increases satiation but has no effect on insulin resistance or the metabolic profile over a 4 day period. Obesity (Silver Spring). 2010;18(6):1176–1182. 10.1038/oby.2009.409 19910942 PMC2998344

[31] Brockman DA, Chen X, Gallaher DD. High-Viscosity Dietary Fibers Reduce Adiposity and Decrease Hepatic Steatosis in Rats Fed a High-Fat Diet. J Nutr. 2014 144(9):1415–22. 10.3945/jn.114.191577 24991042

[32] Maljaars J, Romeyn EA, Haddeman E, Peters HPF, Masclee AAM. Effect of fat saturation on satiety, hormone release, and food intake. Am J Clin Nutr. 2009;89:1019–24. 10.3945/ajcn.2008.27335 19225118

[33] Kozimor A, Chang H, Cooper JA. Effects of dietary fatty acid composition from a high fat meal on satiety. Appetite. 2013;69:39–45. 10.1016/j.appet.2013.05.006 23688821

[34] Poudyal H, Kumar SA, Iyer A, Waanders J, Ward LC, Brown L. Responses to oleic, linoleic and α-linolenic acids in high-carbohydrate, high-fat diet-induced metabolic syndrome in rats. Journal of Nutritional Biochemistry. 2013;24:1381–1392. 10.1016/j.jnutbio.2012.11.006 23333092

[35] Bhaskaran S, Unnikrishnan A, Ranjit R, Qaisar R, Pharaoh G, Matyi S, et al A fish oil diet induces mitochondrial uncoupling and mitochondrial unfolded protein response in epididymal white adipose tissue of mice. Free Radical Biology and Medicine. 2017;108:704–714. 10.1016/j.freeradbiomed.2017.04.028 28455142

[36] Trox J, Vadivel V, Vetter W, Stuetz W, Kammerer DR, Carle R, et al Catechin and epicatechin in testa and their association with bioactive compounds in kernels of cashew nut (Anacardium occidentale L.). Food Chemistry. 2011; 128:1094–1099.

[37] Trox J, Vadivel V, Vetter W, Stuetz W, Scherbaum V, Gola U, et al Bioactive Compounds in Cashew Nut (Anacardium occidentale L.) Kernels: Effect of Different Shelling Methods. J. Agric. Food Chem. 2010;58:5341–5346. 10.1021/jf904580k 20387832

[38] Cardoso BR, Duarte GBS, Reis BZ, Cozzolino SMF. Brazil nuts: Nutritional composition, health benefits and safety aspects. Food Research International. 2017;100:9–18. 10.1016/j.foodres.2017.08.036 28888463

[39] Yang J, Liu RH, Halim L. Antioxidant and antiproliferative activities of common edible nut seeds. LWT—Food Science and Technology. 2009;42:1–8.

[40] Sajilata MG, Singhal RS. Effect of irradiation and storage on the antioxidative activity of cashew nuts. Radiation Physics and Chemistry. 2006;75:297–300.

[41] Barbosa Filho VM, Waczuk EP, Kamdem JP, Abolaji AO, Lacerda SR, Costa JGM, et al Phytochemical constituents, antioxidant activity, cytotoxicity andosmotic fragility effects of Caju (Anacardium microcarpum). Industrial Crops and Products. 2014;55:280–88.

[42] Aguilar YM, Rodríguez FS, Saavedra MA, Espinosa RH, Yero OM. Secondary metabolites and *in vitro* antibacterial activity of extracts from *Anacardium occidentale* L. (Cashew tree) leaves. Revista Cubana de Plantas Medicinales. 2012;17(4):320–329.

[43] Slavin JL. Dietary fiber and body weight. Nutrition. 2005;21:411–418. 10.1016/j.nut.2004.08.018 15797686

[44] Rao TP. Role of guar fiber in appetite control. Physiology & Behavior. 2016;64:277–83. 10.1016/j.physbeh.2016.06.014 27317834

[45] Lovejoy JC, Most MM, Lefevre M, Greenway FL, Rood JC. Effect of diets enriched in almonds on insulin action and serum lipids in adults with normal glucose tolerance or type 2 diabetes. Am J Clin Nutr. 2002;76:1000–1006. 10.1093/ajcn/76.5.1000 12399271

[46] Lee YJ, Nam GE, Seo JA, Yoon T, Seo I, Lee JH, et al Nut consumption has favorable effects on lipid profiles of Korean women with metabolic syndrome. Nutrition Research. 2014;34:814–820. 10.1016/j.nutres.2014.08.011 25238912

[47] Jaiswal YS, Tatke PA, Gabhe SY, Vaidya AB. Antidiabetic activity of extracts of Anacardium occidentale Linn. leaves on n-streptozotocin diabetic rats. Journal of Traditional and Complementary Medicine. 2016;7(4):421–27 10.1016/j.jtcme.2016.11.007 29034189 PMC5634720

[48] Damavandi RD, Eghtesadi S, Shidfar F, Heydari I, Foroushani AR. Effects of hazelnuts consumption on fasting blood sugar and lipoproteins in patients with type 2 diabetes. J Res Med Sci. 2013;18(4):314–321. 24124429 PMC3793377

[49] Sauder KA, Mccrea CE, Kris-Etherton PM, Ulbrecht JS, West SG. Effect of pistachios on lipids, lipoproteins, glucose metabolism, and insulin sensitivity in type 2 diabetes. FASEB Journal 2013;27:368–374. 10.1096/fj.12-213728 23038751

[50] Ma Y, Njike VY, Millet J, Dutta S, Doughty K, Treu JA, et al Effects of Walnut Consumption on Endothelial Function in Type 2 Diabetic Subjects: A randomized controlled crossover trial. Diabetes Care. 2010;33:227–232. 10.2337/dc09-1156 19880586 PMC2809254

[51] Schutte EA, Van Rooyen JM, Huisman HW, Mukuddem-Petersen J, Oosthuizen W, Hanekom SM, et al Modulation of Baroreflex Sensitivity by Walnuts Versus Cashew Nuts in Subjects With Metabolic Syndrome. American Journal of Hypertension 2006;19:629–636. 10.1016/j.amjhyper.2005.12.014 16733237

[52] Almeida-Suhett CP, Graham A, Chen Y, Deuster P. Behavioral changes in male mice fed a high-fat diet are associated with IL-1β expression in specific brain regions. Physiology & Behavior. 2017;169:130–140. 27876639 10.1016/j.physbeh.2016.11.016

[53] Rosqvist F, Iggman D, Kullberg J, Cedernaes J, Johansson HE, Larsson A, et al Overfeeding Polyunsaturated and Saturated Fat Causes Distinct Effects on Liver and Visceral Fat Accumulation in Humans. Diabetes. 2014;63(7):2356–68. 10.2337/db13-1622 24550191

[54] Kahn SE, Cooper ME, Del Prato S. Pathophysiology and treatment of type 2 diabetes: perspectives on the past, presente, and future. The Lancet. 2014;383:1068–1083. 10.1016/S0140-6736(13)62154-6 PMC4226760 24315620

[55] Kahn HS, Valdez R. Metabolic risks identified by the combination of enlarged waist and elevated triacylglycerol concentration. Am J Clin Nutr. 2003;78:928–34. 10.1093/ajcn/78.5.928 14594778

[56] Esmaillzadeh A, Mirmiran P, Azizi F. Clustering of metabolic abnormalities in adolescents with the hypertriglyceridemic waist phenotype. Am J Clin Nutr. 2006;83:36–46. 10.1093/ajcn/83.1.36 16400047

[57] Yang MY, Chan KC, Lee YJ, Chang XZ, Wu CH, Wang CJ. Sechium edule Shoot Extracts and Active Components Improve Obesity and a Fatty Liver That Involved Reducing Hepatic Lipogenesis and Adipogenesis in High-Fat-Diet-Fed Rats. J. Agric. Food Chem. 2015;63:4587−4596. 10.1021/acs.jafc.5b00346 25912298

[58] Vaidya HB, Gangadaran S, Cheema SK. An obesogenic diet enriched with blue mussels protects against weight gain and lowers cholesterol levels in C57BL/6 mice. Nutrition Research. 2017;46:31–37. 10.1016/j.nutres.2017.07.004 29173649

[59] Belchior T, Paschoal VA, Magdalon J, Chimin P, Farias TM, Chaves-Filho AB, et al Omega-3 fatty acids protect from diet induced obesity, glucose intolerance, and adipose tissue inflammation through PPARγ-dependent and PPARγ-independent actions. Mol. Nutr. Food. Res. 2015;59:957–967. 10.1002/mnfr.201400914 25641959

[60] Guo X, Sinclair AJ, Kaur G, Li D. Differential effects of EPA, DPA and DHA on cardio-metabolic risk factors in high-fat diet fed mice. Prostaglandins Leukotrienes and Essential Fatty Acids. 2018;136:47–55. 10.1016/j.plefa.2017.09.011 29113747

[61] Picklo MJ, Idsoa J, Seegerc DR, Aukemad HM, Murphyc EJ. Comparative effects of high oleic acid vs high mixed saturated fatty acid obesogenic diets upon PUFA metabolism in mice. Prostaglandins, Leukotrienes and Essential Fatty Acids. 2017;119:25–37. 10.1016/j.plefa.2017.03.001 28410667

[62] Fabbrini E, Sullivan S, Klein S. Obesity and nonalcoholic fatty liver disease: Biochemical, metabolic, and clinical implications. Hepatology. 2010;51:679–89. 10.1002/hep.23280 20041406 PMC3575093

[63] Lee J, Homma T, Fujii J. Mice in the early stage of liver steatosis caused by a high fat diet are resistant to thioacetamide-induced hepatotoxicity and oxidative stress. Toxicology Letters. 2017;277:92–103. 10.1016/j.toxlet.2017.06.005 28642009

[64] Kristensen M, Jensen MG, Aarestrup J, Petersen KE, Sondergaard L, Mikkelsen MS, et al Flaxseed dietary fibers lower cholesterol and increase fecal fat excretion, but magnitude of effect depend on food type. Nutrition & Metabolism. 2012;3:9–8. 10.1186/1743-7075-9-8 22305169 PMC3307491

[65] Mcrae MP. Dietary fiber is beneficial for the prevention of cardiovascular disease: An umbrella review of meta-analyses. Journal of Chiropractic Medicine. 2017;16(4):289–299. 10.1016/j.jcm.2017.05.005 29276461 PMC5731843

