# Supplementary figures and images for: Cashew nuts (Anacardium occidentale L.) decrease visceral fat, yet augment glucose in dyslipidemic rats
Source: PLoS One. 2019 Dec 12;14(12):e0225736. doi: 10.1371/journal.pone.0225736 (PMC6907795; doi:10.1371/journal.pone.0225736)

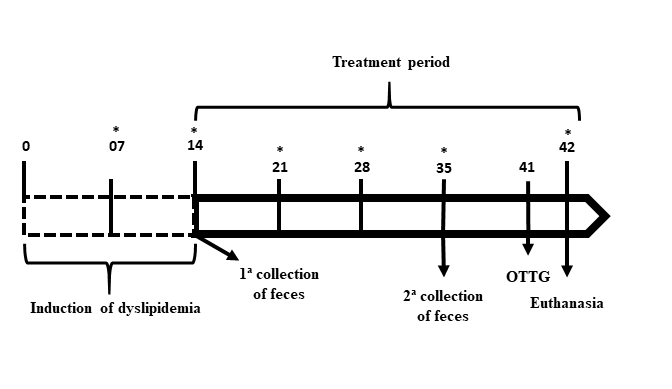

Supplement: S1 Fig — (TIF) [file pone.0225736.s001.tif]

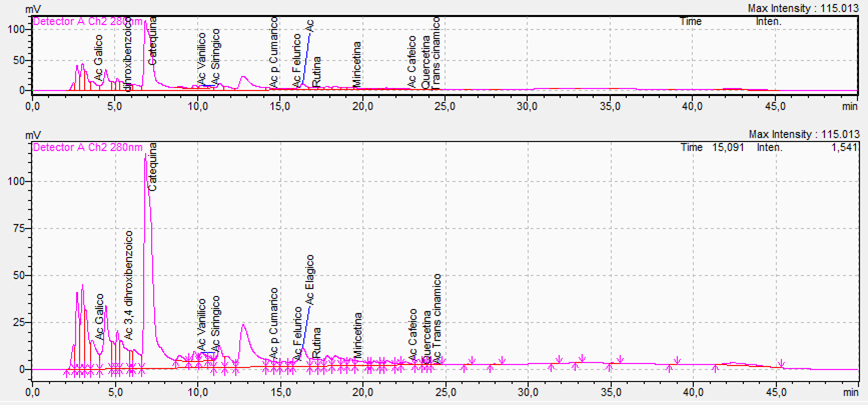

Supplement: S2 Fig — (TIF) [file pone.0225736.s002.tif]
