# Supplementary material for: Cashew nuts (Anacardium occidentale L.) decrease visceral fat, yet augment glucose in dyslipidemic rats
Source: PLoS One. 2019 Dec 12;14(12):e0225736. doi: 10.1371/journal.pone.0225736 (PMC6907795; doi:10.1371/journal.pone.0225736)
Supplement: S1 Table — (DOCX) [file pone.0225736.s003.docx]

**S2 Table.** **Commercial chow composition (Presence Purina ®).**

| **Ingredient** | **Quantities/100g** | **Calories/100g** | **% VCT** |
| --- | --- | --- | --- |
| Protein | 23g | 92 | 24,21% |
| Carbohydrate + Fiber | 63 g | 252 | 66,31% |
| Lipid | 4g | 36 | 9,47% |
| Magnesium | 50 mg | - | - |
| Iron | 18 mg | **-** | - |
| Calcium | 0,3 g | **-** | - |
| Phosphor | 50 mg | **-** | - |
| Sodium | 270 mg | **-** | - |
| Manganese | mg | **-** | - |
| Cobre | 3 mg | **-** | - |
| Zinc | 11 mg | **-** | - |
| Iodine | 0,1 mg | **-** | - |
| Cobalt | 0,2 mg | **-** | - |
| Selenium | 0,02 mg | **-** | - |
| Vitamin A | 2,550 UI | **-** | - |
| Vitamin D3 | 400 UI | **-** | - |
| Vitamin E | 8,2 UI | **-** | - |
| Vitamin K3 | 0,64 mg | **-** | - |
| Vitamin B1 | 1,1 mg | **-** | - |
| Vitamin B2 | 1,2 mg | **-** | - |
| Niacin | 21,9 mg | **-** | - |
| Pantothenic acid | 9 mg | **-** | - |
| Vitamin B6 | 1,1 mg | **-** | - |
| Folic ácid | 1,3 mg | **-** | - |
| Biotin | 0,016 mg | **-** | - |
| Vitamin B12 | 4 mg | **-** | - |
| Choline | 0,25 mg | **-** | - |
| Lysine | 1,25 mg | **-** | - |
| Methionine | 350 mg | **-** | - |
| Tyrosine | 700 mg | **-** | - |
| Gross Energy (Joules) | - | 1.589,9 | 99,99% |
